# Knockdown BMI1 expression inhibits proliferation and invasion in human bladder cancer T24 cells

**DOI:** 10.1007/s11010-013-1745-0

**Published:** 2013-07-03

**Authors:** Wu Liang, Dingjun Zhu, Xuejiang Cui, Jiarui Su, Hongwei Liu, Jinli Han, Fengjin Zhao, Wenlian Xie

**Affiliations:** 1 Department of Urology, The Second Affiliated Hospital of Sun Yat-sen University, No. 107 Yan-jiang West Road, Guangzhou, Guangdong Province 510120 People’s Republic of China; 2 Department of Urology, The Affiliated Hospital of Guangdong Medical College, Zhan-jiang, Guangdong Province People’s Republic of China

**Keywords:** BMI1, Bladder cancer, Invasion, Proliferation, EMT

## Abstract

B cell-specific moloney murine leukemia virus integration site 1 (BMI1) is a transcriptional repressor of polycomb repressive complex 1, which is involved in the proliferation, senescence, migration, and tumorigenesis of cancer. Experimental researchers have convincingly linked BMI1 to tumorigenesis. However, there is no study about the issue on the role of BMI1 in the proliferation, apoptosis, and migration of bladder cancer. To address this question, we examined the expression of BMI1 in bladder cancer tissues and used siRNA to knockdown BMI1 expression in bladder cancer T24 cells. Then we tested the cell proliferation by CCK8 assay and soft agar colony formation assay, apoptosis by flow cytometry assay, and cell invasiveness by transwell migration assay. Our results revealed that BMI1 promoted proliferation, migration, invasion, and progression in bladder cancer. Over-expression of BMI1 was correlated with tumor clinic-pathological features. BMI1 siRNA effectively inhibited bladder cancer cell proliferation and migration in vitro, and it promoted bladder cancer invasion, maybe by causing epithelial-to-mesenchymal transition. Our findings suggested that BMI1 may represent a novel diagnostic marker and a therapeutic target for bladder cancer, and deserves further investigation.

**Electronic supplementary material:**

The online version of this article (doi:10.1007/s11010-013-1745-0) contains supplementary material, which is available to authorized users.

## Introduction

Bladder cancer is a frequent urological malignancy cancer and accounts for about 3 % of all cancer related deaths. In spite of recent progress in its diagnosis and treatment, the molecular mechanisms underlying the development and progression of bladder cancer remain poorly understood, and prognosis of invasive bladder cancer still remains unsatisfactory [1]. Therefore, it is of great urgent to further investigate the molecular mechanisms of bladder cancer metastasis and progression, which will improve the prognosis of invasive bladder cancer.

B-cell-specific moloney murine leukemia virus integration site 1 (BMI1) is a transcriptional repressor of polycomb repressive complex 1(PRC1), which is located at chromosome 10p11.23. It plays an essential role in embryogenesis and maintenance adult stem cell’s self-renewal [2, 3]. BMI1 was originally identified as an oncogene which was associated with c-myc in the generation and development of mouse pre B-cell lymphomas [4]. There is a body of evidences suggesting that BMI1 is involved in the proliferation, senescence, migration, and tumorigenesis of cancer [5–9]. Experimental researchers have convincingly linked BMI1 to tumorigenesis. High expression of BMI1 was associated with aggressive tumor behavior and poor outcome [10].

High expression of BMI1 has been shown to be associated with poor prognosis in bladder cancer [11]. However, the mechanisms of how BMI1 functions to promote bladder cancer progress remain elusive. Because of the significance of BMI1 in cancers tumorigenesis, we hypothesis it may play an important role in bladder cancer tumorigenesis and metastasis. In this study, we investigated the effects of BMI1 knockdown on the proliferation, apoptosis, and invasive in bladder cancer T24 cells. And we found that BMI1 knockdown effectively inhibited bladder cancer T24 cells proliferation and migration in vitro, and it promoted bladder cancer invasion, maybe by causing epithelial-to-mesenchymal transition (EMT).

## Materials and methods

### Patients and samples

To investigate the protein expression, a retrospective review of formalin-fixed, paraffin–embedded tissue sections from 71 patients who underwent transurethral resection, or radical cystectomy, was conducted. Thirty-five normal tissue sections were obtained from the 71 patients at the same time to act as control samples. The protocol for the study was approved by the institutional review board of Sun Yat-sen University. The characteristics of the 71 patients are depicted in Table 1. The use of tumor samples was prospectively approved by the ethics committee of Sun Yat-sen University. Informed consent was obtained from all of the patients.

**Table 1:** Characteristics of archival paraffin-embedded specimens

Adjacent non-cancerous specimens number	35
*Age, years*	
Range	39–83
Mean	65.37 ± 9.69
*Gender*	
Male	30 (85.7)
Female	5 (14.3)
Cancerous specimens number	71
*Age, years*	
Range	34–83
Mean	62.6 ± 10.14
*Gender*	
Male	61 (85.9)
Female	10 (14.1)
*Clinical stage*	
Superficial (T_a_–T_1_)	44 (62.0)
Invasive (T_2_–T_4_)	27 (38.0)
*Pathological grade*	
G_1_	19 (26.8)
G_2_	37 (52.1)
G_3_	15 (21.1)

Values in parentheses are percents

All patients’ histopathological parameters were evaluated according to the 2002 TNM classification of the International Union Against Cancer (UICC) and the International Society of Urological Pathologists (ISUP) consensus classification of urothelial neoplasms of the urinary bladder, which is equivalent to the 2004 WHO grading system.

### Cell culture

Bladder cancer T24 cell was bought from ATCC, and cultured in a humidified CO_2_ incubator at 37 °C in RPMI 1640 (GIBCO), supplemented with 10 % fetal bovine serum and 1 % Penicillin–Streptomycin (Invitrogen).

### Immunohistochemical analysis

4 μm formalin-fixed, paraffin-embedded tissue sections were deparaffinized with xylene, rehydrated in serial dilution of ethanol, and boiled (microwave) for 15 min for antigen retrieval in EDTA buffer (pH 8.0) for BMI1. The sections were then incubated with 3 % hydrogen peroxide for 10 min to quench endogenous peroxidase activity, followed by incubation with goat serum to block nonspecific binding. Slides were washed with PBS and incubated with monoclonal anti-BMI1 antibody (05-637, 1:50, Upstate Biotechnology, Lake Placid, USA) for 1 h at room temperature. They were subsequently reacted with biotinylated secondary antibody for 10 min, followed by incubation with streptavidin-horseradish-peroxidase complex. After further washing, 3,3′-diaminobenzidine (DAB) was applied to the slides as a substrate. Sections were counterstained with Mayer’s hematoxylin, dehydrated with ascending concentrations of alcohol, and mounted by crystal mount. As negative controls, the primary antibody was replaced with non-immune IgG of the same isotype.

The immunohistochemistry scoring was performed by two independent observers. Only nuclear staining was considered positive. Immunoreactivity was classified semi-quantitatively into four categories based on the intensity of staining and the proportion of tumor cells showing unequivocal positive reaction. A staining index (SI) was calculated as a product of staining intensity and area of positive tumor cell nuclei (1, 0–10 % positive cells; 2, 10–50 % positive cells with weak staining intensity; 3, >50 % positive cells with moderate staining intensity; 4, >50 % positive cells with strong staining intensity). In subsequent statistical analyses, the cutoff was based on median SI(<3 vs. ≥3), after considering the frequency distribution curve and size of subgroups.

### Western blot

Briefly, 20ug of Proteins were separated by SDS-PAGE, and transferred to polyvinylidene difluoride (PVDF) membranes (Millipore, Bedford, Massachusetts, USA). membranes were blocked and then probed with antibodies against BMI1 (05-637, 1:150, Upstate Biotechnology, Lake Placid, USA), P16 (BA0266, 1:150, Boster Biological Technology, Wuhan, China), P14 (bs-1174R, 1:250, Bioss Biotechnology, Beijing, China), CDK2 (1:500, abcam), cyclin D1 (1:400, abcam), caspase 3 (1:500, abcam), Bcl-2(1:100, abcam), β-actin (1:1000, abcam), GAPDH (1:1000, abcam). The blots were then washed with tris-buffered saline/tween-20 solution and were incubated with horseradish peroxidase-conjugated goat anti-rabbit IgG (1:5000, Santa cru biotechnology) or goat anti-mouse IgG (1:5000, Santa cru biotechnology) at room temperature. After washed, the blots were exposed using a chemiluminescent detection system (Amersham Life Science, Buckinghamshire, UK).

### BMI1 siRNA and control siRNA transfection

The on-target plus BMI1 pool siRNA and non-targeting control siRNA used for knockdown BMI1 expression were purchased from Santa Cruz Co (sc-29814, California, USA). It is a pool of 3 target-specific 19–25 nt siRNA designed to knock down gene expression [12–14]. They were transfected in T24 cells at a concentration of 100 nm, respectively. T24 cells were cultured at 37 °C in a CO_2_ incubator until the cells were 60–80 % confluent. Subsequently, the cells were transfected with Lipofectamine TM2000 (Invitrogen) according to the manufacturer’s instructions. The transfection reagent (10 μl) and BMI1 siRNA or control siRNA (6 μl each) were incubated with T24 cells in RPMI-1640 culture (serum-free media) for 6 h, and then complete media were used to culture for, a further 48 h, the experiments as described.

### CCK-8 for proliferation assay

Cell proliferation was assessed using WST-8 dye (Beyotime institute of biotechnology, China), according to the manufacturer’s instructions/protocol. Briefly, 5,000 T24 cells/wells were seeded in a 96-well cell culture plate, grown at 37 °C for 24 h, and then placed in serum-started condition for another 6 h. Subsequently, T24 cells were transfected with BMI1 siRNA and control siRNA, respectively. On the day of measuring the growth rate of untreated and transfected cells, 100 μl of spent medium was replaced with an equal volume of fresh medium containing 10 % CCK8, then cells continued to be incubated at 37 °C for 3 h, and the absorbance was finally determined at 450 nm using a micro plate reader.

### Soft agar colony formation assay

Briefly, 6-well plates were coated with a bottom layer of 1.5 ml base agar consisting of 0.5 % agar, 1*RPMI 1640 medium, and 10 % fetal bovine serum, followed by a top layer of 1 ml 0.35 % agar. In each well, 5000 cells were plated over the top layer. Each assay was performed in triplicate wells. Plates were assessed for number and size of colonies under a microscope after 3 weeks of incubation. All experiments were performed three times.

### Flow cytometry assay

After infection treatment, cells in each well were harvested, and cell apoptosis was determined by annexin, using the V-FITC/PI staining method. Tests were performed in triplicate for each sample, and analyses were performed by FAC-Scan flow cytometry (Becton–Dickinson, San Jose, USA), according to the manufacturer’s guidelines.

### In vitro migration assays

The migration in vitro assays were performed using 24-well transwell units (Corning, New York, USA) with an 8-μm-pore-size polycarbonate filter. Cells that migrated to the lower surface of the filter were counted under a light microscope at a magnification of 400×, and the number of migratory cells was counted.

### Statistical analysis

Data analyses were performed using SPSS 11.0 (SPSS Inc., Chicago, USA).and data were expressed as mean ± SD. The differences between groups were compared using Student’s *t* test, and differences between populations based on positive or negative IHC results were analyzed by χ^2^ test, the relationship between BMI1 expression, and clinic-pathologic features were also analyzed by χ^2^ test. All *p* value <0.05 was considered statistically significant.

## Results

### BMI1 was over-expressed in bladder cancer tissue than corresponding normal adjacent bladder tissues

**Fig. 1:**
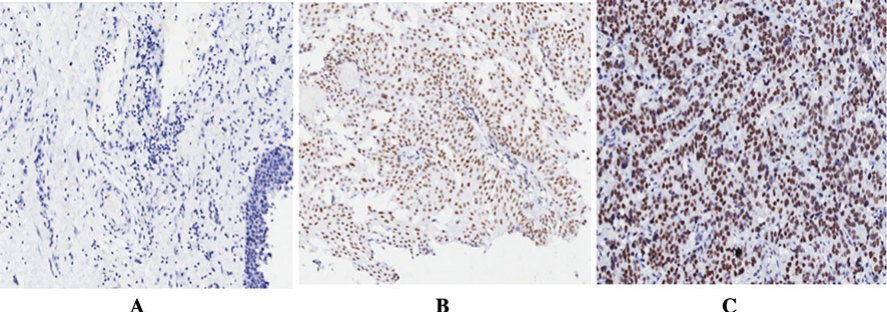
Analysis of BMI1 expression by immunohistochemistry in normal bladder, non-invasive bladder cancer, and invasive bladder cancer tissues. BMI1 expression was localized within the nuclei. **a** None BMI1 staining in Normal bladder tissue, **b** moderate staining in non-invasive bladder cancer tissue, **c** strong staining in invasive bladder cancer tissue (original magnification ×200)

BMI1 protein expression was analyzed by Immunohistochemistry. IHC staining showed that 53.5 % (38/71) of bladder cancer tissues were moderately or strongly positive for BMI1 staining, which was expressed in the neoplastic epithelial cell nuclei. This compared with 20.0 % (7/35) staining in the adjacent normal tissue samples. (*p* < 0.05) (Fig. 1). And the staining was higher in bladder cancer tissues than the adjacent normal tissues.

### BMI1 expression was associated with the clinic-pathological features of bladder cancer

The relationship between the clinic-pathological features and the BMI1 expression is shown in Table 2. There was no significantly different between BMI1 protein expression and patients’ age and gender and number of tumors. However, the protein expression was significantly, positively associated with, tumor size, clinical stage, and pathological grade.

**Table 2:** Relationship of BMI-1 protein expression with clinicopathological characteristics of bladder carcinoma

Item	Cases	BMI-1 expression (%)		χ^2^	*p* value
		Negative	Positive		
*Gender*				0.010	0.919
Male	61	29 (47.5)	32 (52.5)		
Female	10	4 (40.0)	6 (60.0)		
*Age (years)*				0.051	0.821
≤50	9	5 (55.6)	4 (44.4)		
>50	62	28 (45.2)	34 (54.8)		
*Tumor size (cm)*				5.431	0.020*
≤3 in diameter	39	23 (59.0)	16 (41.0)		
>3 in diameter	32	10 (31.3)	22 (68.7)		
*Tumor number*				0.558	0.455
Single	53	26 (49.1)	27 (50.9)		
≥2	18	7 (38.9)	11 (61.1)		
*Clinical stage*				17.560	*p* < 0.001*
Superficial (T_is_, T_a_, T_1_)	44	29 (65.9)	15 (34.1)		
Invasive (T_2_, T_3_, T_4_)	27	4 (14.8)	23 (85.2)		
*Pathological grade*				10.036	0.007*
G1	19	14 (73.7)	5 (26.3)		
G2	37	16 (43.2)	21 (56.8)		
G3	15	3 (20.0)	12 (80.0)		

* represents statistical significance, *p* < 0.05

### BMI1 suppressed the expression of p16 and p14 in bladder cancer T24 cells

**Fig. 2:**
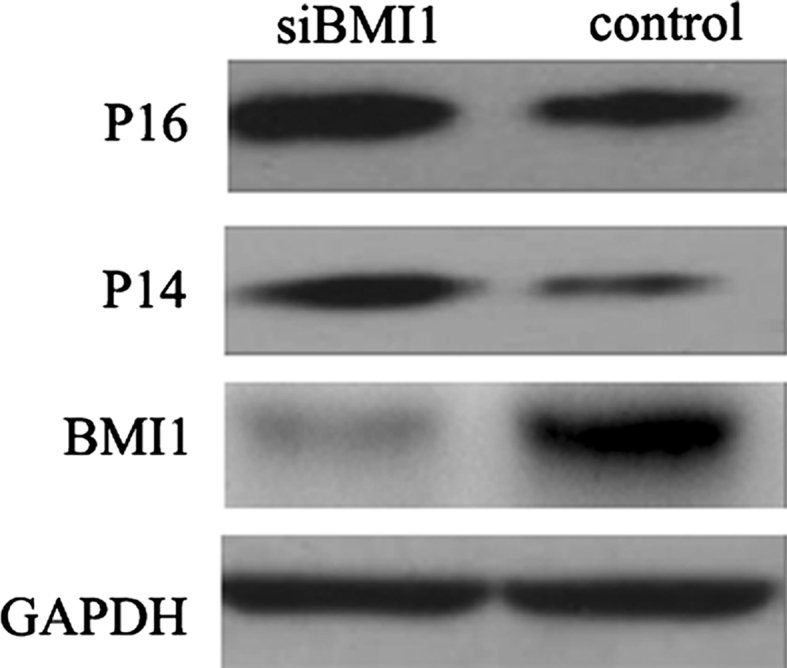
Effect of BMI1 knockdown on the expression of BMI1, P16, and P14 in bladder cancer T24 cells, measured by western blot. The BMI1 was knockdown by using siRNA transfected. After BMI1 knockdown, the expression of P16 and P14 increased

BMI1 has been proved to suppress the transcription of p16 and p14 in many tumors. To determine whether BMI1 suppresses the expression of p16 and p14 in bladder cancer, we used siRNA to silence BMI1 expression, and then detected the expression of p16 and p14. Western blot showed that p16 and p14 expression was increased after BMI1 knockdown (Fig. 2).

### BMI1 knockdown inhibited T24 cells growth and proliferation

**Fig. 3:**
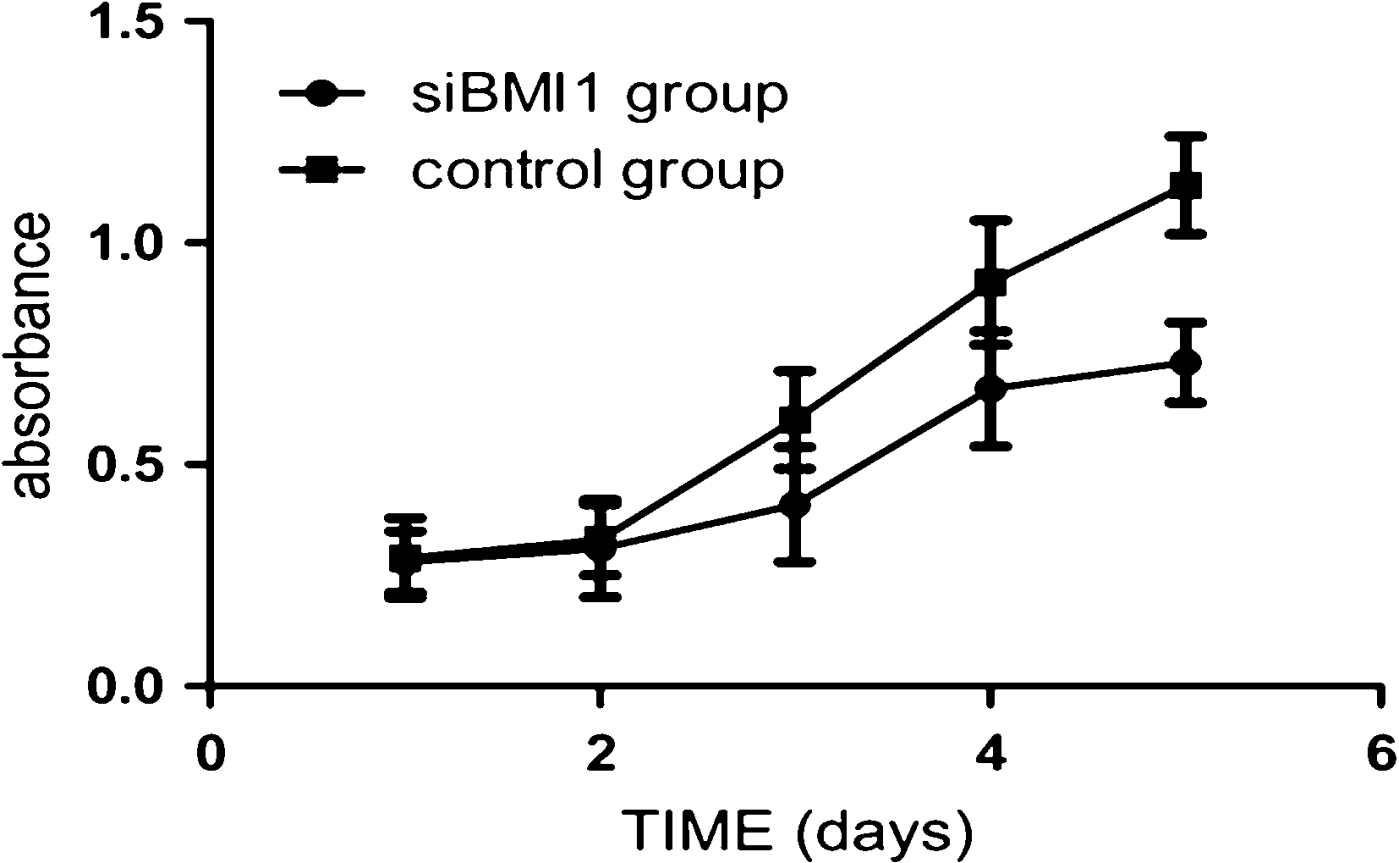
BMI1 knockdown inhibited the proliferation of T24 cells in vitro. The growth rates in BMI1 knockdown group was significantly reduced than control group, measured by CCK8 assay. The growth curves were determined in triplicate, and they were representative of three independent experiments

**Fig. 4:**
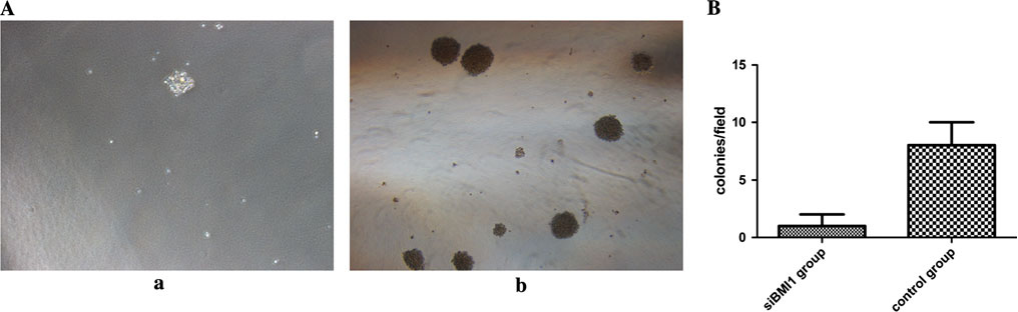
**a** Soft agar colony formation assay showed that BMI1 knockdown reduced T24 cell colony formation. Colonies larger than 1 mm in diameter were counted. The number of T24 colonies was significantly reduced by BMI1 knockdown. (*a*) BMI1 siRNA group (*b*) control group. **b** The *bar graph* showed the mean number of colonies in 10 low power fields for each group. *p* < 0.05, Student *t* test

To determine the role of BMI1 in cancer cells, we transduced BMI1 siRNA into bladder cancer T24 cells. Then we examined the effect of silencing BMI1 on cell viability using CCK-8 assays. We observed a slight inhibition in growth at 2 days after transduction. Furthermore, obvious inhibitory effects on cell proliferation were observed in BMI1 silenced cells at 3–5 days (Fig. 3, *p* < 0.05). The soft agar colony formation assay also showed that the numbers of colonies in BMI1 knockdown group was fewer and smaller than the control group (Fig. 4, *p* < 0.05).

### BMI1 knockdown prompted T24 cells apoptosis

**Fig. 5:**
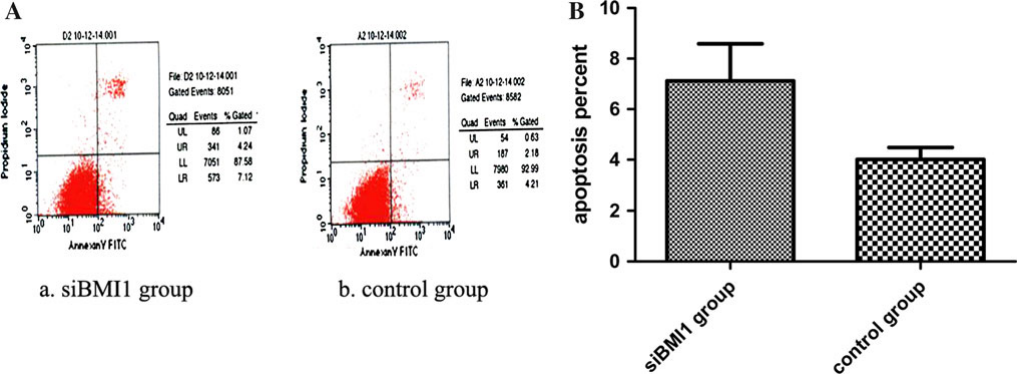
**a** BMI1 knockdown enhanced the apoptosis of T24 cells. Apoptotic cells of different groups were measured by flow cytometry after 48 h transduction. The cell populations of Annexin-V +/PI− and Annexin-V +/PI+ were used to assess apoptotic events. (*a*) BMI1 siRNA group, (*b*) control group. **b** The apoptosis rate of BMI1 siRNA group cells was significantly higher than that of control group cells. *p* < 0.05, Student *t* test

**Fig. 6:**
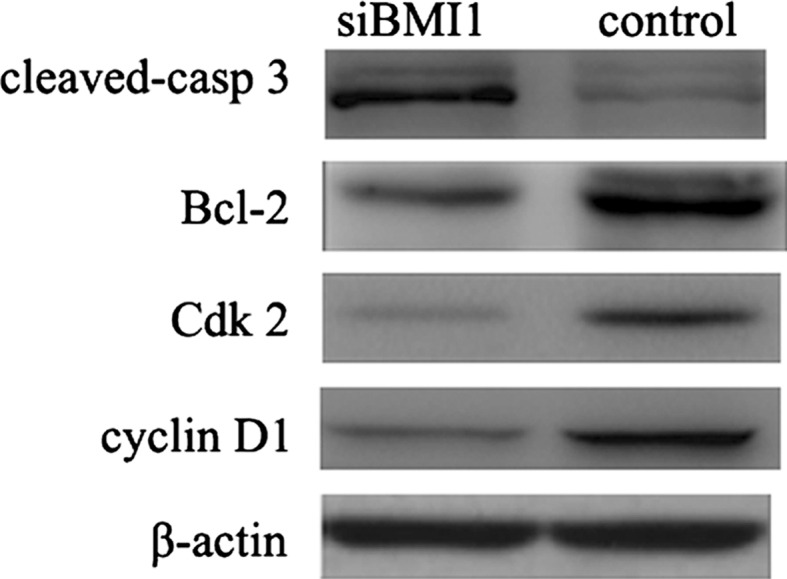
Effect of BMI1 knockdown on the expression of cell cycle and apoptosis related genes, measured by western blot. Cyclin D1, cdk2, BCL2 were decreased in BMI1 siRNA group cells than control cells, while apoptosis protein cleaved-caspase3 was increased

Flow cytometry showed that a marked increase in apoptosis was observed in BMI1 siRNA group cells, compared to control cells after 48 h transduction. The ratio of apoptosed cells (Annexin-V+/PI- and Annexin-V+/PI+) in BMI1 depleted cells was significantly higher than in control cells (Fig. 5). WB showed that cell cycle and anti-apoptosis related genes cyclin D1, cdk2, Bcl-2 were decreased in BMI1 siRNA group cells than control cells, while apoptosis protein cleaved-caspase3 was increased (Fig. 6).

### BMI1 knockdown decreased T24 cells migration in vitro

**Fig. 7:**
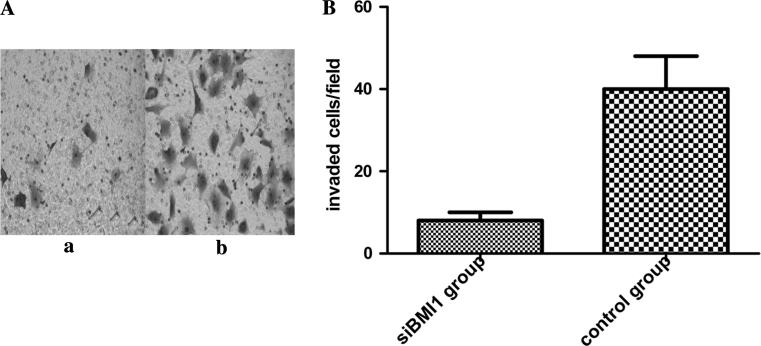
**a** Effect of BMI1 siRNA on the migration of T24 cells in vitro. A.Transwell migration assay was performed to measure T24 cell migration. Crystal violet staining of the lower surface filters showed that the cells were able to migrate to the filter (×400). The number of the invaded cells of the control group is dramatically more than that of the BMI1 siRNA group. (*a*) BMI1 siRNA group, (*b*) control group. **b** Quantification of the numbers of BMI1 siRNA group and control group cells migrating through the matrigel. *p* < 0.05, Student *t* test

Because BMI1 was higher expression in invasive bladder cancer than in non-invasive bladder cancer, we hypothesized that BMI1 regulated the invasive and migration abilities of bladder cancer. To investigate this hypothesis, we measured the effect of BMI1 on T24 cells using an in vitro transwell migration assay. We observed that the number of migrated cells decreased significantly in BMI1 knockdown cells, compared with that of control cells (Fig. 7, *p* < 0.05).

### BMI1 siRNA reduced the migration capacity of T24 cells through EMT change

**Fig. 8:**
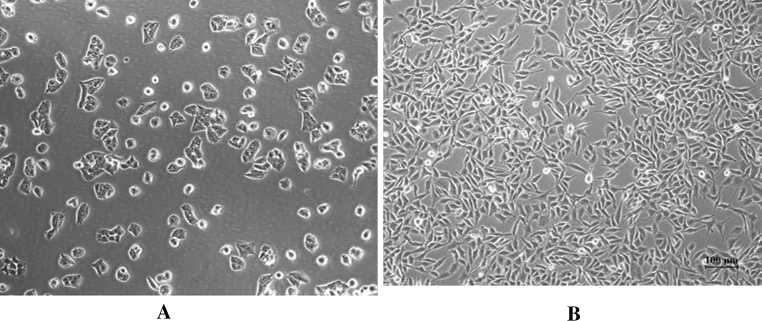
Morphologic changes in T24 cells after BMI1 knockdown. **a** BMI1 siRNA group, **b** control group. In the BMI1 siRNA group, cells showed cobblestone-like morphology, whereas the control group cells kept their fibroblastic, elongated like morphology. Photos were taken under ×200 magnification

**Fig. 9:**
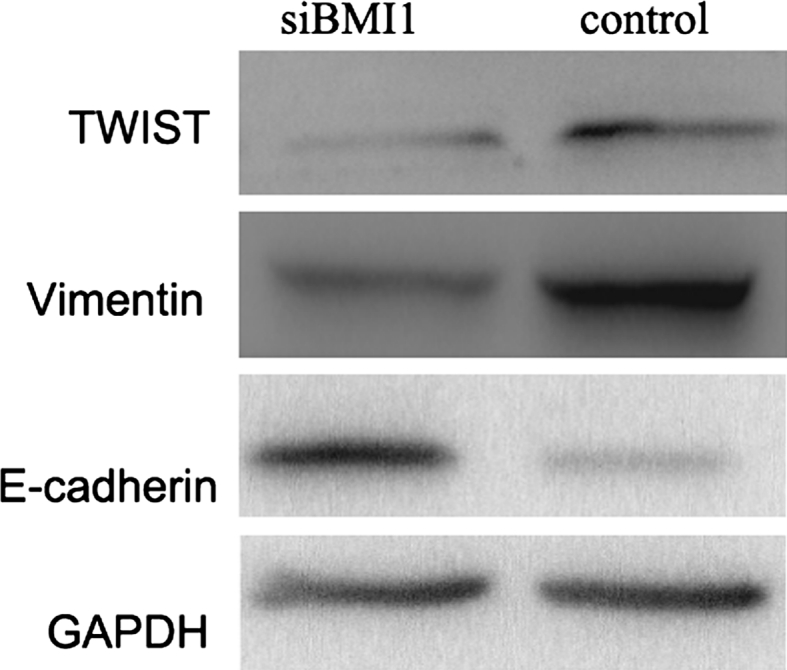
Effect of BMI1 knockdown on the expression of epithelial marker E-cadherin and mesenchymal marker vimentin and twist, measured by western blot. The epithelial marker E-cadherin was increased, whereas the mesenchymal marker vimentin and twist decreased in BMI1 siRNA group cells than control group cells

In our study, we found that the morphology of cells change obviously after BMI1 knockdown. In BMI1 siRNA group cells showed cobblestone-like phenotype, whereas the control group cells kept their fibroblastic, elongated like morphology (Fig. 8). To find out whether the effect of BMI1 knockdown on T24 cells migration was associated with EMT, we examined the expression of epithelial marker E-cadherin and mesenchymal marker vimentin by western blot. The result showed that the epithelial marker E-cadherin increased, whereas the mesenchymal marker vimentin decreased in BMI1 siRNA group cells (Fig. 9). These findings suggested that BMI1 increased cells migration maybe through EMT change.

## Discussion

In this study, we found that BMI1 was highly expressed in bladder cancer and its expression was correlated with clinic-pathological features. We demonstrated here that BMI1 promoted bladder cancer cell proliferation, migration, and progression by inhibition p16 and p14 expression. Knockdown of endogenous BMI1 expression reduced bladder cancer proliferation and invasiveness.

PcG protein expression is often up-regulated in many types of human cancers. BMI1 is a member of the PRC1 complex; it controls many diverse biological cancer processes including differentiation, senescence, proliferation, migration, and tumorigenesis [15]. Much evidence have linked BMI1 to aggressive, malignant behavior, and poor clinical outcome in various cancer cells, including prostate cancer, breast cancer, lung cancer, ovarian cancer, and hepatocellular cancer [16–19]. Regarding the relationship between BMI1 expression and bladder cancer clinic-pathology, there are very few reports about their relationship. A prior study found that BMI1 was highly expressed in bladder cancer, and served as a prognostic marker in bladder cancer, supporting our results on BMI1 expression in bladder cancer [11]. However, they are unclear as to the molecular mechanism of how BMI1 influences bladder cancer development, how BMI1 promotes bladder cancer proliferation and migration. It has been proved that ink-14-arf is the downstream target of BMI1, which encodes two tumor suppressor proteins, p16 and p14. The p16-pRb pathway and p53-p14 pathway are two tumor suppressor pathways that are inactivated in many cancers [20–23].

Over-expression of BMI1 or deletions of p16 and p14 are frequently discovered in many cancers, but their roles in bladder cancer have not been previously recognized. We have demonstrated here, that by using siRNA to silence BMI1 expression, the p16 and p14 expression are increased, which in turn inhibited cell growth in bladder cancer. These results suggested that BMI1 promoted bladder cancer proliferation by inhibiting the p16-pRB and p53-p14 pathways. These results were similar to studies performed on other cancers that BMI1 promoted cancer cell proliferation through inhibition of ink-14-arf locus expression. However, it was also well known that BMI1 modulated the DDR pathway and PI3K/AKT pathway that then caused cancer cell progression [24, 25]. So, the precise molecular mechanism of BMI1 in the tumorigenesis and proliferation of bladder cancer needs to be further explored.

Additionally, it was reported that BMI1 was involved in the metastasis of cancers. Over-expression of BMI1 was associated with increased risk of metastasis [26, 27]. Here, our results indicated that BMI1 expression displayed correlation with the clinic-pathological features of the bladder cancers, and BMI1 expression in invasive bladder cancer was significantly higher than that in superficial bladder cancer. Furthermore, BMI1 siRNA in bladder cancer T24 cells reduced the migration of bladder cancer in vitro. These results provided powerful evidence that silencing BMI1 expression contributed to a reduction in the migration of bladder cancer, and suggested that BMI1 was significantly associated with the metastatic ability of bladder cancer.

Epithelial-to-mesenchymal transition is a process by which cancer cells change their epithelial phenotype into a mesenchymal phenotype and acquire metastatic ability, which is closely associated with the invasive and metastatic process of cancers [28]. Many reports have addressed the association between BMI1 and EMT. A study has shown that BMI1 inhibited the expression of PTEN, and modulated PI3K/AKT/GSK-3b/snail signaling, which promotes EMT and enhances the metastatic ability of nasopharyngeal cancer cells [25]. Another study demonstrated that twist1 regulated BMI1 expression, which then resulted in the occurrence of EMT and cancer stem cell characteristics [29]. In our study, Consistent with the reduced metastatic behavior of these cancer cells, the epithelial marker E-cadherin increased in BMI1 siRNA group cells, whereas the mesenchymal marker vimentin and twist decreased. Combined with our study, all these supported the idea that BMI1 promoted the invasion and migration of bladder cancer maybe through acquiring EMT characteristics. However, the exact mechanism requires further investigation.

In conclusion, our results have demonstrated that BMI1 promoted proliferation, migration, invasion, and progression in bladder cancer. Over-expression of BMI1 was correlated with tumor clinic-pathological features. BMI1 siRNA effectively inhibited bladder cancer cell proliferation and migration in vitro, and it promoted bladder cancer invasion, maybe by causing EMT. Our findings suggest that BMI1 may represent a novel diagnostic marker and a therapeutic target for bladder cancer, and deserves further investigation.

## Electronic supplementary material

Below is the link to the electronic supplementary material.

Supplementary material 1 (PDF 64 kb)
