# Supplementary material for: Knockdown BMI1 expression inhibits proliferation and invasion in human bladder cancer T24 cells
Source: Mol Cell Biochem. 2013 Jul 3;382(1):283–91. doi: 10.1007/s11010-013-1745-0 (PMC3771375; doi:10.1007/s11010-013-1745-0)
Supplement: Supplementary file 1 — Supplementary material 1 (PDF 64 kb) [file 11010_2013_1745_MOESM1_ESM.pdf]

SANTA CRUZ BIOTECHNOLOGY, INC.

Bmi-1 siRNA (h): sc-29814

BACKGROUND

In *Drosophila*, Polycomb (Pc-G) gene family encodes chromatin proteins that are required for the repression of homeotic loci in embryonic development. Mel-18 and Bmi-1 are mammalian homologs of *Drosophila* Pc-G group proteins, as they are similarly expressed during development and implicated in the regulation of gene expression and axial skeleton development, as well as the control of proliferation and survival of haematopoietic cells. Mel-18 directly binds to DNA through a RING-finger motif and preferentially associates with juxtaposed enhancer elements on various genes, including Bcl-2, c-Myc and Hox. Mel-18 is an immediate early response gene within the c-Myc/Cdc25 signaling cascade that exhibits tumor suppressor activity and negatively regulates cell cycle progression by blocking S phase entry. Alternatively, Bmi-1 has been identified as a potent oncogene as it contributes to the transcriptional activation of genes implicated in early lymphoid development. Proviral activation of Bmi-1 expression corresponds to enhanced gene-specific activation of other proto-oncogenes, including c-Myc and Pim, subsequently resulting in the progression of lymphomagenesis.

REFERENCES

1. Tagawa, M., et al. 1990. Expression of novel DNA-binding protein with zinc finger structure in various tumor cells. J. Biol. Chem. 265: 20021-20026.
2. Goebl, M.G. 1991. The Bmi-1 and Mel-18 gene products define a new family of DNA-binding proteins involved in cell proliferation and tumorigenesis. Cell 66: 623.

CHROMOSOMAL LOCATION

Genetic locus: BMI1 (human) mapping to 10p11.23.

PRODUCT

Bmi-1 siRNA (h) is a pool of 3 target-specific 19-25 nt siRNAs designed to knock down gene expression. Each vial contains 3.3 nmol of lyophilized siRNA, sufficient for a 10 µM solution once resuspended using protocol below. Suitable for 50-100 transfections. Also see Bmi-1 shRNA Plasmid (h): sc-29814-SH and Bmi-1 shRNA (h) Lentiviral Particles: sc-29814-V as alternate gene silencing products.

For independent verification of Bmi-1 (h) gene silencing results, we also provide the individual siRNA duplex components. Each is available as 3.3 nmol of lyophilized siRNA. These include: sc-29814A, sc-29814B and sc-29814C.

STORAGE AND RESUSPENSION

Store lyophilized siRNA duplex at -20° C with desiccant. Stable for at least one year from the date of shipment. Once resuspended, store at -20° C, avoid contact with RNAses and repeated freeze thaw cycles.

Resuspend lyophilized siRNA duplex in 330 µl of the RNAse-free water provided. Resuspension of the siRNA duplex in 330 µl of RNAse-free water makes a 10 µM solution in a 10 µM Tris-HCl, pH 8.0, 20 mM NaCl, 1 mM EDTA buffered solution.

RESEARCH USE

For research use only, not for use in diagnostic procedures.

APPLICATIONS

Bmi-1 siRNA (h) is recommended for the inhibition of Bmi-1 expression in human cells.

SUPPORT REAGENTS

For optimal siRNA transfection efficiency, Santa Cruz Biotechnology's siRNA Transfection Reagent: sc-29528 (0.3 ml), siRNA Transfection Medium: sc-36868 (20 ml) and siRNA Dilution Buffer: sc-29527 (1.5 ml) are recommended. Control siRNAs or Fluorescein Conjugated Control siRNAs are available as 10 µM in 60 µl. Each contain a scrambled sequence that will not lead to the specific degradation of any known cellular mRNA. Fluorescein Conjugated Control siRNAs include: sc-36869, sc-44239, sc-44240 and sc-44241. Control siRNAs include: sc-37007, sc-44230, sc-44231, sc-44232, sc-44233, sc-44234, sc-44235, sc-44236, sc-44237 and sc-44238.

GENE EXPRESSION MONITORING

Bmi-1 (H-99): sc-10745 is recommended as a control antibody for monitoring of Bmi-1 gene expression knockdown by Western Blotting (starting dilution 1:200, dilution range 1:100-1:1000) or immunofluorescence (starting dilution 1:50, dilution range 1:50-1:500).

To ensure optimal results, the following support (secondary) reagents are recommended: 1) Western Blotting: use goat anti-rabbit IgG-HRP: sc-2004 (dilution range: 1:2000-1:100,000) or Cruz Marker™ compatible goat anti-rabbit IgG-HRP: sc-2030 (dilution range: 1:2000-1:5000), Cruz Marker™ Molecular Weight Standards: sc-2035, TBS Blotto A Blocking Reagent: sc-2333 and Western Blotting Luminol Reagent: sc-2048. 2) Immunofluorescence: use goat anti-rabbit IgG-FITC: sc-2012 (dilution range: 1:100-1:400) or goat anti-rabbit IgG-TR: sc-2780 (dilution range: 1:100-1:400) with UltraCruz™ Mounting Medium: sc-24941.

DATA

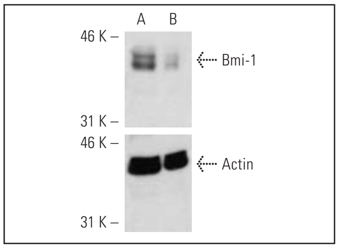

Bmi-1 siRNA (h): sc-29814. Western blot analysis of Bmi-1 expression in non-transfected control (**A**) and Bmi-1 siRNA transfected (**B**) HeLa cells. Blot probed with Bmi-1 (C-20): sc-8906. Actin (I-19): sc-1616 used as specificity and loading control.

RT-PCR REAGENTS

Semi-quantitative RT-PCR may be performed to monitor Bmi-1 gene expression knockdown using RT-PCR Primer: Bmi-1 (h)-PR: sc-29814-PR (20 µl, 471 bp). Annealing temperature for the primers should be 55-60° C and the extension temperature should be 68-72° C.
